# The Effect of Corporate Governance and Transformational Leadership on Business Strategy and Business Performance Moderated by Government Policy

**DOI:** 10.12688/f1000research.175209.2

**Published:** 2026-04-20

**Authors:** Muhammad Willy, Mochammad Al Musadieq, Yusri Abdillah, Nila Firdausi Nuzula

**Affiliations:** 1 Brawijaya University, Malang, East Java, Indonesia

**Keywords:** Corporate Governance, Transformational Leadership, Business Strategy, Business Performance, Government Policy, PLS-SEM

## Abstract

**Background:**

The performance of regional-owned multi-business enterprises (BUMD Aneka Usaha) in Indonesia is crucial for regional economic development, yet many still face challenges related to weak corporate governance, leadership, and business strategy.

**Methods:**

Data were collected through a questionnaire survey administered to 176 directors of regional-owned multi-business enterprises across Indonesia. The data were analysed using Partial Least Squares Structural Equation Modelling (PLS-SEM), with higher-order constructs estimated in SmartPLS.

**Results:**

The findings show that corporate governance and transformational leadership have positive and significant effects on business strategy and business performance. Business strategy also has a positive and significant effect on business performance and mediates the effects of corporate governance and transformational leadership on performance. Government policy significantly moderates the relationships between corporate governance, transformational leadership, business strategy, and business performance, strengthening their positive impact.

**Conclusions:**

These results highlight the need to reinforce internal capabilities through stronger governance practices, transformational leadership, and flexible, innovation-oriented strategies, while ensuring a supportive government policy environment so that regional-owned enterprises can improve their business performance.

## 1. Introduction

In the current era of accelerating globalization, organizations operate in increasingly dynamic and uncertain environments, characterised by rapid technological change, intensifying competition, and evolving stakeholder expectations. ^1, 2^ Firms are required not only to survive but also to sustain and enhance their performance over time in order to remain relevant. In emerging economies, public enterprises face an additional layer of complexity because they are expected to achieve both commercial and social objectives. ^3^ In Indonesia, Regional-Owned Enterprises ( *Badan Usaha Milik Daerah*, BUMD) constitute a key policy instrument for local governments to support regional development and generate locally sourced revenue ( *Pendapatan Asli Daerah*, PAD). ^4^

Business performance is therefore a critical outcome for BUMD, as it reflects the extent to which these entities are able to fulfil their financial and non-financial mandates. ^5^ The business performance construct is inherently multidimensional, encompassing indicators such as profitability, revenue growth, market share, customer satisfaction, innovation, and long-term sustainability. ^6, 7^ In the context of BUMD, performance is assessed not only in terms of financial returns to local governments, but also in terms of their contribution to public welfare through reliable services and efficient use of public resources. ^8, 9^ Empirical evidence, however, shows that many BUMD contribute only modestly to PAD, face recurring financial deficits, and are frequently classified as financially “distressed” or “unhealthy”. These conditions suggest that a substantial proportion of BUMD have not yet been able to translate their mandates and resource endowments into satisfactory business performance.

Therefore, business strategy becomes a central lever through which BUMD can enhance their performance. A well-formulated and well-implemented strategy provides direction for resource allocation, market positioning, and organizational priorities in responding to environmental change. ^9, 10^ Building on the Resource-Based View (RBV) and Dynamic Capabilities Theory, prior studies argue that firms achieve superior performance when they are able to configure and reconfigure internal resources to create unique value, adapt to regulatory and market shifts, and innovate in products, services, and processes. ^11– 13^ In the BUMD context, however, evidence suggests that many enterprises still lack clear, adaptive business strategies or roadmaps, leading to inefficiencies, limited responsiveness to external changes, and suboptimal performance. ^14, 15^ Strengthening business strategy is therefore essential for improving both the efficiency and effectiveness of BUMD operations.

Within the RBV perspective, internal organizational factors such as corporate governance and transformational leadership represent critical strategic capabilities that shape how business strategies are formulated and executed. Corporate governance provides the structures, processes, and control mechanisms that promote transparency, accountability, fairness, responsibility, and board independence, thereby reducing agency problems and supporting sound strategic decision-making. ^16– 18^ Empirical studies show that robust corporate governance is associated with better strategic alignment and value creation. ^19, 20^ Transformational leadership, in turn, enables leaders to articulate a compelling vision, inspire and motivate followers, stimulate critical thinking, and attend to individual needs, which is particularly important for overcoming bureaucratic inertia and driving organizational change in public enterprises. ^21^ Prior research has documented positive effects of transformational leadership on strategic decision quality and firm performance, often through mediating mechanisms such as innovation and corporate social responsibility. ^22– 25^ Nevertheless, the joint role of corporate governance and transformational leadership in shaping business strategy and performance in the specific setting of Indonesian BUMD remains underexplored.

In addition to internal factors, government policy also plays an important role in determining the direction and effectiveness of BUMD performance. ^26, 27^ Rigid regulations, complex licensing bureaucracy, and weak supervisory functions can slow down strategic decision-making and reduce organizational flexibility. Conversely, supportive policies may strengthen governance, clarify strategic direction, and facilitate leadership that is oriented toward transformation. Government policy therefore has the potential to act as a moderating variable that either reinforces or weakens the relationships among these variables.

Existing empirical studies have largely examined the relationships between business strategy and performance, between corporate governance and performance, or between transformational leadership and performance in private firms, listed companies, banks, or central state-owned enterprises, and mainly in developed or other emerging economies. ^28, 29^ Only a limited number of studies focus on BUMD, and even fewer consider how internal capabilities interact with the external policy environment. In practice, BUMD operate under dense regulatory frameworks, where government policy can either constrain or enable strategic and managerial choices. However, the moderating role of government policy in the links between corporate governance, transformational leadership, business strategy, and business performance has not been systematically investigated in the context of Indonesian Regional-Owned Enterprises.

Based on the above considerations, this study aims to examine the effects of corporate governance and transformational leadership on business strategy and business performance, with business strategy positioned as a mediating mechanism linking internal capabilities to business performance, while taking into account the role of government policy as a moderating variable. This study is expected to fill the research gap that remains limited in the BUMD context and to provide a theoretical contribution through the application of RBV. In addition, the study contributes by clarifying the strategic role of business strategy as a mediating mechanism, extending the application of RBV, DCT, and Contingency Theory in the public enterprise context, and offering practical insights for strengthening governance, leadership, and policy support to improve business performance.

## 2. Literature review

### 2.1 Theoretical review

The Resource-Based View (RBV) explains organizational performance by focusing on internal resources and capabilities rather than external market conditions. It posits that firms achieve sustainable competitive advantage when they possess resources that are valuable, rare, difficult to imitate, and not easily substituted by competitors. ^11, 30, 31^ These resources may include tangible assets, human capital, organizational routines, and managerial capabilities that are embedded in the firm’s structures and processes. ^32^ RBV also recognises that firms are heterogeneous in their resource endowments, which leads to differences in strategic options and performance outcomes across organizations. Later developments extend RBV to consider network and ecosystem resources, showing that jointly used or relational assets can further enhance competitive advantage in complex environments. ^33, 34^

Contingency Theory provides a complementary perspective by arguing that there is no single best way to organise or lead an organisation, and that effectiveness depends on the fit between internal arrangements and external conditions. Early formulations emphasised the match between leadership style and situational factors such as task structure, leader–member relations, and positional power. ^35, 36^ Subsequent work extended the theory to organisational design and strategy, suggesting that structures, processes, and strategic choices must be aligned with environmental characteristics, including market dynamics, technology, and regulatory frameworks. ^37^ In this view, internal practices such as governance, leadership, and strategy do not operate in isolation but are conditioned by the policy and institutional context in which organisations operate. Contingency Theory therefore offers a useful lens for understanding how government policy can strengthen or weaken the performance effects of internal capabilities in public and hybrid organisations such as regional-owned enterprises. ^38^

### 2.2 Corporate governance

Corporate governance is an effort to maximize corporate profits in a manner that does not impose undue costs on other individuals or on society as a whole. ^16^ According to Jeżak and Bohdanowicz, ^39^ corporate governance is the way in which a company resolves conflicts among different groups that influence corporate management, thereby shaping its strategy and behavior in the capital market, the goods and services market, and the labour market. Ayuso and Argandoña ^40^ define corporate governance as a system by which organizations are directed and controlled, which determines the distribution of rights and responsibilities between shareholders and managers, as well as the rules and procedures for decision-making in corporate matters. In a broader sense, corporate governance also encompasses relationships with other stakeholders, both internal (e.g., employees) and external (e.g., customers, suppliers). ^19^

### 2.3 Transformational leadership

Transformational leadership is a leadership style that fosters positive change in individuals and organizations. ^41^ It is a mechanism through which leaders and employees promote virtue, enthusiasm, inspiration, and determination in pursuing, acquiring, and sharing knowledge to achieve common goals, while setting aside personal interests. ^22^ Within transformational leadership, leaders act more as agents of change by demonstrating role-model behavior and inspiring their members to prioritize collective interests over individual ones. ^21^

### 2.4 Business strategy

Business strategy is the approach used by firms to achieve long-term objectives and optimize performance in a continuously changing environment. ^2^ To adapt to shifts in markets, technology, and regulation, firms must possess the capability to undergo ongoing transformation. Teece et al. ^12^ explains that firms must be able to innovate and manage change in order to remain competitive. These dynamic capabilities include the ability to identify external opportunities or threats, adapt business strategies, and deploy resources more effectively to address emerging challenges. ^12^

### 2.5 Business performance

Business performance refers to the extent to which a firm or organization is able to achieve its predetermined objectives, taking into account various dimensions such as operational efficiency, strategic effectiveness, and the attainment of desired outcomes. ^42^ Business performance encompasses multiple aspects that go beyond financial results, including product quality, customer satisfaction, as well as sustainability and adaptability to market changes. ^43^

Business performance can thus be understood as a measure of how far a firm is able to reach its targets in terms of growth, profitability, and sustainability within a competitive environment. ^44^ It also includes how the firm utilizes existing resources to create value, adapt to market changes, and sustain its competitive advantage. ^45^ For regionally owned enterprises, knowledge of governance and effective management is crucial for their success and for contributing to local economic development. ^46^

### 2.6 Government policy

Government policy is defined as the actions taken by the government to achieve particular objectives. According to Jenkins, ^47^ government policy is a set of decisions made by political actors to choose goals and the means to achieve those goals. Government policy can be understood as whatever government chooses to do or not to do. ^48^ Thus, government policy constitutes actions or activities undertaken or deliberately not undertaken by the government in pursuit of national objectives. Such policies include laws, government regulations, political decisions, programs, priorities, budgets, and so forth, which are formulated to address public problems or meet societal needs. ^49^ Accordingly, government policy has a substantial impact on the lives of citizens.

## 3. Methods

**Figure 1.**
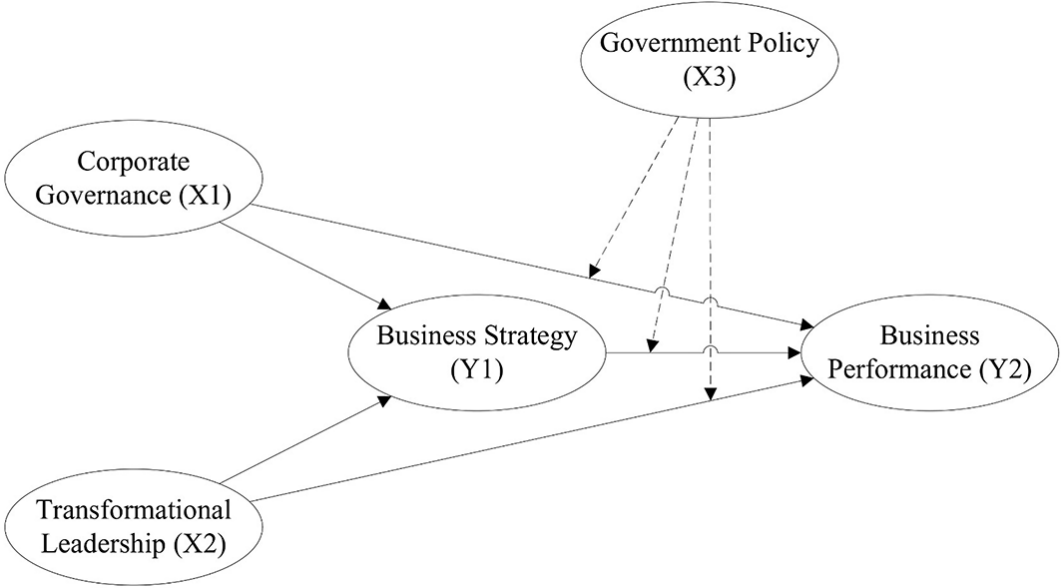
Research Framework.

This study employed a quantitative, explanatory research design using a cross-sectional survey approach. The design was used to examine the direct, mediating, and moderating relationships among corporate governance, transformational leadership, business strategy, government policy, and business performance in regional-owned multi-business enterprises (BUMD *Aneka Usaha*) in Indonesia. Data were analysed using SmartPLS, a software application for Partial Least Squares Structural Equation Modeling (PLS-SEM). SmartPLS was selected because it is suitable for handling complex models with multiple constructs and relationships, as well as small to medium sample sizes. ^50^ This software enables the evaluation of both the measurement model and the structural model, thereby ensuring construct validity and reliability and testing the hypothesised relationships among variables, as presented in Figure 1.

### 3.1 Data collection

The data for this study were collected through a structured questionnaire administered to 176 respondents. The respondents consisted of members of the Board of Directors of each BUMD Aneka Usaha, with each company represented by at least two individuals. According to data from the Ministry of Home Affairs in 2023, there are 313 BUMD Aneka Usaha owned by provincial and district/municipal governments across Indonesia. ^51^ Thus, the population of this study comprised 313 BUMD *Aneka Usaha*.

Given resource constraints, this study employed a sampling technique. The method used was random sampling, which provides each member of the population with an equal probability of being selected as part of the sample. This approach allows the collected data to remain relevant and comprehensive, while enhancing the reliability and validity of the research findings. To implement random sampling, the list of 313 BUMD *Aneka Usaha* was first compiled based on the Ministry of Home Affairs data. The sample units were then selected randomly from this population list, so that each enterprise had an equal chance of being included in the study.

### 3.2 Indicators of variables

The indicators used in this study were carefully selected to ensure comprehensive measurement of the key research variables, namely Corporate Governance, Transformational Leadership, Business Strategy, Business Performance, and Government Policy. The selection of indicators was based on prior literature and considerations of relevance to the context of this study. Accordingly, each variable was measured through a set of indicators that not only reflect theoretical aspects but also capture practical dimensions pertinent to the conditions of BUMD Aneka Usaha in Indonesia. The detailed indicators for each research variable are presented systematically in Table 1.

**Table 1.** Indicators of each variable.

Variable	Indicator	Source
Corporate Governance (X1)	Transparency (X1.1)	^16, 39, 40, 52– 54^
	Accountability (X1.2)	
	Fairness (X1.3)	
	Responsibility (X1.4)	
	Board Independence (X1.5)	
Transformational Leadership (X2)	Idealized Influence (X2.1)	^23, 24, 55, 56^
	Inspirational Motivation (X2.2)	
	Intellectual Stimulation (X2.3)	
	Individualized Consideration (X2.4)	
Government Policy (X3)	Policy Effectiveness (X3.1)	^51, 57– 60^
	Policy Stability (X3.2)	
	Inclusiveness (X3.3)	
	Operational Support (X3.4)	
	Compatibility (X3.5)	
Business Strategy (Y1)	Product/Service Innovation and Adaptation (Y1.1)	^9, 12^
	Organizational Flexibility and Decision-Making (Y1.2)	
	Resource Allocation and Management (Y1.3)	
Business Performance (Y2)	Financial Returns (Y2.1)	^1, 28, 29, 61, 62^
	Operational Excellence (Y2.2)	
	Market Performance. (Y2.3)	
	Profitability (Y2.4)	
	Sales Growth (Y2.5)	
	Customer Satisfaction (Y2.6)	
	Company Reputation (Y2.7)	

## 4. Results

In this study, inferential analysis was conducted using Partial Least Squares Structural Equation Modelling (PLS-SEM). Consistent with the conceptual and operational definitions of the variables, the model was specified as a hierarchical component model (second-order), in which each higher-order construct is represented by several lower-order dimensions. The analysis was carried out with SmartPLS, which is suitable for recursive models, accommodates latent variables with indicators, and allows the estimation of moderation effects and higher-order constructs.

### 4.1 Measurement model

In line with the embedded two-stage approach for higher-order constructs, the lower-order constructs (LOCs) were first assessed and found to meet the recommended thresholds for reliability and validity; therefore, the following results focus on the measurement model at the higher-order construct (HOC) level. The HOC measurement model was evaluated in terms of indicator reliability, internal consistency reliability, and convergent validity. Table 2 presents the standardized outer loadings, Cronbach’s alpha, composite reliability, and Average Variance Extracted (AVE) for all higher-order constructs.

**Table 2.** Indicator reliability, internal consistency reliability, and convergent validity at the higher-order construct level.

Variable	Indicators	*Outer Loadings*	*Cronbach’s Alpha*	*Composite Reliability*	AVE
Corporate Governance (X1)	X1.1	0.822	0.875	0.870	0.657
	X1.2	0.840			
	X1.3	0.788			
	X1.4	0.778			
	X1.5	0.822			
Transformational Leadership (X2)	X2.1	0.850	0.876	0.867	0.809
	X2.2	0.854			
	X2.3	0.855			
	X2.4	0.856			
Government Policy (X3)	X3.1	0.814	0.888	0.842	0.613
	X3.2	0.799			
	X3.3	0.800			
	X3.4	0.713			
	X3.5	0.785			
Business Strategy (Y1)	Y1.1	0.876	0.816	0.862	0.784
	Y1.2	0.891			
	Y1.3	0.889			
Business Performance (Y2)	Y2.1	0.778	0.858	0.881	0.586
	Y2.2	0.727			
	Y2.3	0.802			
	Y2.4	0.783			
	Y2.5	0.831			
	Y2.6	0.739			
	Y2.7	0.888			

Source: Processed Primary Data (2025).

As shown in Table 2, all standardized outer loadings are above the recommended threshold of 0.708, indicating that each indicator contributes substantially to its respective higher-order construct. For example, the indicators of Corporate Governance (X1) exhibit outer loadings ranging from 0.778 to 0.840, while Transformational Leadership (X2) shows loadings between 0.850 and 0.856. Business Strategy (Y1) presents loadings in the range of 0.876–0.891, Business Performance (Y2) between 0.727 and 0.888, and Government Policy (X3) between 0.713 and 0.814, supporting adequate indicator reliability across all constructs.

Internal consistency reliability was assessed using Cronbach’s alpha and composite reliability. All constructs report values greater than 0.70, which indicates acceptable to high reliability. For instance, Transformational Leadership (X2) records a Cronbach’s alpha of 0.876 and a composite reliability of 0.867, while Business Performance (Y2) shows values of 0.858 and 0.881, respectively. Corporate Governance (X1), Business Strategy (Y1), and Government Policy (X3) likewise meet or exceed the recommended cut-off values, confirming that the indicators within each higher-order construct are internally consistent.

Convergent validity was evaluated using the Average Variance Extracted (AVE). All constructs achieve AVE values above 0.50, suggesting that each higher-order construct explains more than 50% of the variance in its indicators. Transformational Leadership (X2) has an AVE of 0.809, Business Strategy (Y1) 0.784, and Government Policy (X3) 0.613, while Corporate Governance (X1) and Business Performance (Y2) also display AVE values exceeding 0.50. These results indicate that convergent validity is satisfactorily established for all higher-order constructs.

The next criterion to be considered is discriminant validity. This validity is assessed using the Heterotrait–Monotrait (HTMT) ratio, which serves as a benchmark for examining the extent to which the constructs in the model are empirically distinct from one another. Table 3 reports the HTMT values among the higher-order constructs.

**Table 3.** HTMT values at the higher-order construct level.

	X1	X2	X3	Y1	Y2
**X1**					
**X2**	0.641				
**X3**	0.512	0.533			
**Y1**	0.491	0.538	0.634		
**Y2**	0.557	0.673	0.684	0.656	

Source: Processed Primary Data (2025).

All HTMT values are below the conservative threshold of 0.90, with, for example, a value of 0.641 between Corporate Governance (X1) and Transformational Leadership (X2), 0.656 between Business Strategy (Y1) and Business Performance (Y2), and 0.684 between Government Policy (X3) and Business Performance (Y2). These results indicate that the constructs are empirically distinct from one another, thereby confirming discriminant validity at the HOC level.

Taken together, the evidence for indicator reliability, internal consistency reliability, convergent validity, and discriminant validity shows that the measurement model at the higher-order level is robust and suitable for subsequent structural model evaluation and hypothesis testing.

### 4.2 Structural model evaluation

In PLS-SEM, the evaluation of the structural model is conducted to identify and assess the relationships among the latent variables. The present study follows the procedures recommended by Hair et al. (2022). The first step involves examining potential collinearity issues in the structural model, as high collinearity may bias the estimation of path coefficients. Collinearity was assessed using the Variance Inflation Factor (VIF), which detects the extent to which the independent variables are linearly related to each other.

As shown in Table 4, all VIF values are well below the threshold of 3.3 suggested by Kock (2015), indicating that collinearity is not a concern in this model. Corporate Governance (X1) has a VIF of 1.350 and Transformational Leadership (X2) a VIF of 1.010, both of which suggest that there is no significant collinearity with other variables. Business Strategy (Y1) and Business Performance (Y2) report VIF values of 1.738 and 1.795, respectively, which remain within acceptable limits and indicate low levels of collinearity. Similarly, Government Policy (X3) has a VIF value of 1.693, also below the critical cut-off. Overall, the VIF values for all variables fall within the acceptable range, suggesting that there is no significant multicollinearity among the independent variables. Consequently, the estimated path coefficients in the structural model can be regarded as reliable and not distorted by collinearity problems.

**Table 4.** VIF values for each variable.

Variable	VIF
Corporate Governance (X1)	1.350
Transformational Leadership (X2)	1.010
Government Policy (X3)	1.693
Business Strategy (Y1)	1.738
Business Performance (Y2)	1.795

Source: Processed Primary Data (2025).

### 4.3 Hypothesis testing

**4.3.1 Direct effects**

The analysis of direct effects in this study involves two exogenous variables (Corporate Governance and Transformational Leadership), one endogenous mediating construct (Business Strategy), and one final endogenous construct (Business Performance). The results of the direct effect analysis are presented in Table 5.

**Table 5.** Results of direct effect testing.

Hypothesis	Relationship between Variables	Path coefficient	* p-value *	Conclusion
	Independent variable ➔ Dependent variable			
H1	Corporate Governance (X1) ➔ Business Strategy (Y1)	0.431	0.000	Significant
H2	Transformational Leadership (X2) ➔ Business Strategy (Y1)	0.240	0.031	Significant
H3	Business Strategy (Y1) ➔ Business Performance (Y2)	0.262	0.001	Significant
H4	Corporate Governance (X1) ➔ Business Performance (Y2)	0.206	0.005	Significant
H5	Transformational Leadership (X2) ➔ Business Performance (Y2)	0.175	0.040	Significant

Source: Processed Primary Data (2025).

The following paragraphs provide a more detailed explanation of each direct effect tested in this study.

H1: **Corporate Governance (X1) has an effect on Business Strategy (Y1)**

The effect of Corporate Governance (X1) on Business Strategy (Y1) yields a path coefficient of 0.431 with a p-value of 0.000. Since the p-value is less than 0.05 and the coefficient is positive, Corporate Governance has a positive and significant effect on Business Strategy. This indicates that better corporate governance practices are associated with more effective business strategies. Thus, hypothesis 1 is supported.

H2: **Transformational Leadership (X2) has an effect on Business Strategy (Y1)**

Transformational Leadership (X2) has a path coefficient of 0.240 with a p-value of 0.031 in its effect on Business Strategy (Y1), indicating a positive and significant relationship. This suggests that higher-quality transformational leadership contributes to the development and implementation of better business strategies. Hence, hypothesis 2 is supported.

H3: **Business Strategy (Y1) has an effect on Business Performance (Y2)**

Business Strategy (Y1) exhibits a path coefficient of 0.262 with a p-value of 0.001 in its effect on Business Performance (Y2). This result demonstrates that Business Strategy exerts a positive and significant influence on Business Performance, indicating that it serves as an important mediating mechanism through which internal organizational capabilities are translated into improved performance outcomes. Therefore, hypothesis 3 is supported.

H4: **Corporate Governance (X1) has an effect on Business Performance (Y2)**

Corporate Governance (X1) shows a positive and significant direct effect on Business Performance (Y2), with a path coefficient of 0.206 and a p-value of 0.005. This finding indicates that sound corporate governance directly contributes to enhanced business performance. Thus, hypothesis 4 is supported.

H5: **Transformational Leadership (X2) has an effect on Business Performance (Y2)**

Transformational Leadership (X2) also has a positive and significant effect on Business Performance (Y2), with a path coefficient of 0.175 and a p-value of 0.040. This suggests that transformational leadership directly supports improvements in business performance. Therefore, hypothesis 5 is supported.

**4.3.2 Indirect effects**

Business Strategy (Y1) serves as a mediating variable that links Corporate Governance (X1) and Transformational Leadership (X2) to Business Performance (Y2). By including Business Strategy as a mediator, the indirect effects of the exogenous variables on the endogenous variable can be identified. The results of the mediation analysis are presented in Table 6.

**Table 6.** Results of indirect effect testing.

Hypothesis	Variable			Path coefficient	*p-value*	Conclusion
	Independent variable	Mediating variable	Dependent variable			
H6	Corporate Governance (X1)	Business Strategy (Y1)	Business Performance (Y2)	0.113	0.006	Significant
H7	Transformational Leadership (X2)	Business Strategy (Y1)	Business Performance (Y2)	0.063	0.035	Significant

Source: Processed Primary Data (2025).

As shown in Table 6, Corporate Governance (X1) exerts a positive and significant indirect effect on Business Performance (Y2) through Business Strategy (Y1), with a path coefficient of 0.113 and a p-value of 0.006. This implies that improvements in corporate governance enhance business performance partly through the strengthening of business strategies.

**Table 7.** Results of moderation effect testing.

Hypothesis	Independent variable ➔ Dependent variable	Path coefficient	*p-value*	Conclusion
H8	Corporate Governance (X1) × Government Policy (X3) ➔ Business Performance (Y2)	0.161	0.039	Significant
H9	Transformational Leadership (X2) × Government Policy (X3) ➔ Business Performance (Y2)	0.115	0.025	Significant
H10	Business Strategy (Y1) × Government Policy (X3) ➔ Business Performance (Y2)	0.168	0.040	Significant

Source: Processed Primary Data (2025).

Similarly, Transformational Leadership (X2) has a positive and significant indirect effect on Business Performance (Y2) via Business Strategy (Y1), with a path coefficient of 0.063 and a p-value of 0.035. This finding indicates that transformational leadership facilitates the development of more effective business strategies, which in turn lead to better performance outcomes. Overall, Business Strategy is confirmed as a significant mediating variable in the relationships between the exogenous variables and Business Performance.

**4.3.3 Moderating effects of government policy**

The moderating role of Government Policy (X3) was examined to determine the extent to which government policy strengthens or weakens the effects of Corporate Governance (X1), Transformational Leadership (X2), and Business Strategy (Y1) on Business Performance (Y2). The results of the moderation analysis are summarised in Table 7.

H8: **Government Policy (X3) moderating the effect of Corporate Governance (X1) on Business Performance (Y2)**

The interaction between Corporate Governance (X1) and Government Policy (X3) on Business Performance (Y2) yields a path coefficient of 0.161 with a p-value of 0.039. This indicates a positive and significant moderating effect, suggesting that a more supportive government policy environment strengthens the impact of corporate governance on business performance. Thus, hypothesis 8 is supported.

H9: **Government Policy (X3) moderating the effect of Transformational Leadership (X2) on Business Performance (Y2)**

The interaction between Transformational Leadership (X2) and Government Policy (X3) on Business Performance (Y2) results in a path coefficient of 0.115 with a p-value of 0.025. This demonstrates that Government Policy significantly enhances the positive effect of Transformational Leadership on Business Performance. Therefore, hypothesis 9 is supported.

H10: **Government Policy (X3) moderating the effect of Business Strategy (Y1) on Business Performance (Y2)**

The interaction between Business Strategy (Y1) and Government Policy (X3) on Business Performance (Y2) shows a path coefficient of 0.168 with a p-value of 0.040. This implies that supportive government policy amplify the positive effect of Business Strategy on Business Performance. Hence, hypothesis 10 is supported.

**4.3.4 Predictive relevance of the model (PLSpredict)**

To evaluate the predictive relevance of the model, the PLSpredict procedure was employed. This procedure compares the predictive performance of the PLS-SEM model with that of a benchmark linear model (LM) using Q ^2^predict, Root Mean Square Error (RMSE), and Mean Absolute Error (MAE). The results are presented in Table 8.

**Table 8.** PLSpredict results.

	PLS			LM	
	Q ^2^predict	RMSE	MAE	RMSE	MAE
**Y1.1**	0.097	0.930	0.751	0.957	0.778
**Y1.2**	0.136	0.906	0.747	0.935	0.795
**Y1.3**	0.104	0.904	0.728	0.953	0.762
**Y2.1**	0.281	0.854	0.721	0.873	0.720
**Y2.2**	0.220	0.889	0.732	0.882	0.730
**Y2.3**	0.259	0.866	0.726	0.944	0.759
**Y2.4**	0.198	0.900	0.747	0.903	0.720
**Y2.5**	0.281	0.854	0.701	0.861	0.710
**Y2.6**	0.171	0.916	0.781	0.944	0.806
**Y2.7**	0.137	0.935	0.775	0.965	0.803

Source: Processed Primary Data (2025).

All Q ^2^predict values are greater than zero (ranging from 0.097 to 0.281), indicating that the model exhibits meaningful predictive relevance. Positive Q ^2^predict values suggest that the PLS-SEM model outperforms a naïve benchmark model without predictors. In terms of predictive accuracy, the RMSE and MAE values for the PLS model are generally lower or very close to those of the LM benchmark. For several indicators (e.g. Y1.1, Y1.2, Y1.3, Y2.3, Y2.6, Y2.7), both RMSE and MAE for PLS are lower than those for LM, indicating that the PLS model provides slightly more accurate predictions. Overall, the combination of positive Q ^2^predict values and RMSE/MAE metrics that are comparable to or better than those of the LM benchmark indicates that the PLS-SEM model has adequate predictive power and can be considered reliable for predicting indicators of Business Strategy and Business Performance in new data.

## 5. Discussion

### 5.1 The effect of corporate governance on business strategy

The results indicate that corporate governance has a positive and significant effect on business strategy, with a path coefficient of 0.431 and a p-value of 0.000. This supports H1 and shows that stronger corporate governance practices are associated with more effective business strategies in regional-owned enterprises. Corporate governance in this study is reflected by dimensions such as transparency, accountability, fairness, responsibility, and board independence, while business strategy is formed by product and service innovation, organizational flexibility, and the allocation and management of resources.

These findings are consistent with RBV, which posits that sustainable competitive advantage stems from valuable, rare, inimitable, and well-organized internal resources. ^11, 63^ In this context, an effective governance system functions as a strategic capability that shapes how decisions are made, risks are managed, and resources are allocated. Transparent and accountable governance processes provide a robust framework for strategic decision-making, enabling organizations to respond more quickly and coherently to environmental changes.

From a DCT perspective, corporate governance is relevant because it helps organizations align and reconfigure internal resources in response to changing conditions. ^12^ In regional-owned enterprises, governance mechanisms such as accountability, transparency, and board independence support strategic adjustment, organizational flexibility, and innovation. The significant effect found in this study therefore suggests that corporate governance contributes not only as an internal capability under RBV, but also as a driver of strategic adaptation as emphasized by DCT.

The results corroborate previous studies by Singh, ^17^ Saltaji, ^19^ Yakimov, ^20^ and Al-Azzam et al., ^64^ which consistently demonstrate that sound corporate governance positively influences strategic management processes. Principles such as transparency, accountability, and board independence offer safeguards against opportunistic behaviour and help align strategic decisions with long-term organizational sustainability. This is particularly salient for publicly monitored entities such as regional-owned enterprises, where strategic decisions must be both effective and accountable.

At the indicator level, accountability and transparency emerge as the strongest governance dimensions, suggesting that clear lines of responsibility and open information disclosure are especially critical for shaping strategic choices. ^65^ On the strategy side, organizational flexibility is the most dominant dimension, indicating that good governance tends to be associated with more agile structures and processes, which are essential for timely adaptation in complex and regulated markets. ^2^

In practice, regional-owned enterprises operate across diverse sectors such as trade, services, mining, and transport, which inherently increases managerial complexity. In such a context, governance becomes a necessary foundation to maintain strategic coherence across multi-business portfolios. Overall, the findings underscore that strengthening corporate governance is not merely a compliance requirement but a strategic imperative. Governance mechanisms that emphasize transparency, accountability, and board independence provide the structural and procedural conditions needed to develop innovative products and services, enhance organizational flexibility, and allocate resources efficiently—ultimately supporting more robust and sustainable business strategies.

### 5.2 The effect of transformational leadership on business strategy

The analysis shows that transformational leadership has a positive and significant effect on business strategy, with a path coefficient of 0.240 and a p-value of 0.031, thereby supporting H2. Transformational leadership in this study is reflected by idealized influence, inspirational motivation, intellectual stimulation, and individualized consideration. Among these, individualized consideration provides the strongest contribution, indicating that leaders’ attention to individual needs, aspirations, and development is particularly influential in shaping overall leadership quality.

Business strategy is represented by product and service innovation, organizational flexibility, and resource allocation and management, with organizational flexibility again emerging as the strongest dimension. This pattern suggests that a leadership style that emphasizes individual development and support tends to foster a more adaptive, flexible, and innovative strategic posture. ^66^ From an RBV perspective, transformational leadership can be seen as an intangible, firm-specific resource that enhances the deployment of other resources and capabilities. ^63^ By articulating a compelling vision, stimulating new ways of thinking, and attending to the personal growth of employees, transformational leaders help translate internal resources into coherent and adaptive strategic initiatives.

Transformational leadership is also relevant to DCT because it helps mobilize organizational change and foster learning-oriented responses to evolving demands. ^12^ In this study, the significant effect of transformational leadership on business strategy suggests that leadership strengthens the organization’s capacity to renew strategic direction, encourage initiative, and support ongoing improvement. This indicates that transformational leadership contributes not only as an internal capability, but also as a catalyst for strategic renewal under DCT.

The findings are consistent with previous research by Yasmeen et al. ^25^ and Abuzaid et al., ^67^ which demonstrate that transformational leadership significantly influences strategy formulation and implementation. Through inspirational motivation and intellectual stimulation, transformational leaders create a supportive climate for questioning existing routines, exploring new strategic options, and embracing change.

The prominence of individualized consideration in this study is particularly noteworthy. Leaders who mentor, coach, and recognise employees as individuals foster higher levels of trust, commitment, and discretionary effort. ^68^ These conditions make employees more willing and able to support strategic changes, thereby enhancing the organization’s adaptability. In line with the RBV logic, transformational leadership operates as a valuable internal capability that leverages human capital to create adaptive, hard-to-imitate business strategies. ^63^

Overall, the positive and significant effect of transformational leadership on business strategy indicates that leadership quality is a critical lever for building and maintaining adaptive strategic capabilities. In the context of regional-owned enterprises, where organizations face both commercial pressures and public mandates, transformational leadership supports the development of strategies that are not only innovative and flexible but also aligned with broader organizational and societal goals.

### 5.3 The effect of business strategy on business performance

Business strategy has a positive and significant effect on business performance, with a path coefficient of 0.262 and a p-value of 0.001, thereby confirming H3. Business strategy in this study comprises product and service innovation, organizational flexibility, and resource allocation and management, while business performance is measured through financial returns, operational excellence, market performance, profitability, sales growth, customer satisfaction, and company reputation.

Within the RBV framework, strategy represents the way an organization orchestrates its internal resources and capabilities to achieve superior performance. Strategies that emphasize innovation, flexibility, and efficient resource allocation enable firms to adjust to environmental changes and exploit emerging opportunities more effectively. ^63^ The strong role of organizational flexibility underscores that the ability to reconfigure structures and processes quickly is central to performance enhancement in dynamic environments.

Business strategy is relevant to DCT because it provides the organizational direction through which adaptive responses can be translated into concrete performance outcomes. ^12^ In this study, the significant effect of business strategy on business performance indicates that strategies emphasizing innovation, flexibility, and disciplined resource use enable regional-owned enterprises to respond more effectively to changing demands. This suggests that business strategy functions not only as a planning instrument, but also as a practical vehicle for sustaining performance under DCT.

These results are in line with Handoyo et al., ^14^ Ricardianto et al., ^15^ Arif et al., ^18^ and Ilmudeen and Bao, ^1^ who find that effective business strategies significantly improve firm performance. In this study, organizational flexibility emerges as the dominant strategic dimension, which logically connects to key performance indicators such as market performance, sales growth, and company reputation. The capacity to adapt quickly to regulatory changes, political priorities, and market dynamics appears to be a decisive factor in converting strategic intent into actual performance gains.

Differences with Latifah et al. ^28^ study, which report non-significant direct effects, may be attributed to sectoral and contextual variation. Regional-owned enterprises operate in complex, highly regulated environments where strategic misalignment can have immediate consequences, making the direct impact of strategy on performance more visible. In less regulated or smaller-scale contexts, the effect of strategy may be more indirect and mediated by other capabilities, such as innovation or information systems.

Overall, the findings suggest that developing and implementing flexible, innovation-oriented strategies is crucial for enhancing the performance of regional-owned enterprises. The prominence of organizational flexibility indicates that the capacity to adapt quickly to environmental change is a key performance driver across financial, operational, and reputational dimensions.

### 5.4 The effect of corporate governance on business performance

H4, which posits that corporate governance affects business performance, is supported, with a path coefficient of 0.206 and a p-value of 0.005. This confirms a positive and significant relationship between corporate governance and the performance of regional-owned enterprises. Corporate governance in this study is measured through transparency, accountability, fairness, responsibility, and board independence. These elements collectively contribute to improvements across all dimensions of business performance, including financial outcomes, operational efficiency, market position, growth, customer satisfaction, and reputation.

From an RBV standpoint, effective governance can be interpreted as an organizational capability that enables better coordination, control, and alignment between stakeholders and strategic goals. ^63^ Transparent reporting and clear accountability reduce information asymmetry and agency problems, thereby enhancing decision quality and stakeholder trust.

From a DCT perspective, corporate governance is also relevant because it helps organizations maintain strategic responsiveness by supporting the alignment and reconfiguration of resources in changing environments. ^12^ In regional-owned enterprises, governance mechanisms such as accountability, transparency, and board independence can strengthen the organization’s ability to adjust priorities, improve operational discipline, and respond more effectively to regulatory and market demands. The significant direct effect found in this study therefore suggests that corporate governance contributes not only as an internal capability under RBV, but also as a mechanism that supports organizational adaptation and sustained performance under DCT.

The findings align with prior research by Nag and Chatterjee, ^69^ Mustafa et al., ^18^ and Çelik and Gülle, ^70^ which show that strong governance structures improve economic performance and firm value. In this study, accountability emerges as the strongest governance dimension. When roles, responsibilities, and performance expectations are clearly defined and enforced, managers and directors are more likely to deploy resources efficiently, minimise waste, and avoid misuse, thereby directly supporting operational excellence and profitability.

Overall, the results provide strong evidence that corporate governance is a key lever for improving the business performance of regional-owned enterprises. Strengthening accountability, transparency, fairness, responsibility, and board independence is essential not only for compliance but also for building long-term competitive and reputational advantages in the public enterprise context.

### 5.5 The effect of transformational leadership on business performance

H5, which examines the effect of transformational leadership on business performance, is supported. The path coefficient is 0.175 with a p-value of 0.040, indicating a positive and significant relationship. Transformational leadership, reflected by idealized influence, inspirational motivation, intellectual stimulation, and individualized consideration, shows consistently significant loadings, confirming the robustness of the construct. Among these dimensions, individualized consideration provides the strongest contribution. This highlights the importance of leaders who invest in employees’ personal development, provide tailored support, and acknowledge individual differences.

Through the RBV lens, transformational leadership can be considered a unique and valuable intangible resource that is difficult for competitors to imitate. ^63^ By elevating employee motivation, fostering psychological safety, and encouraging innovation, transformational leaders help convert the potential of human resources into superior performance at both operational and financial levels. The results are consistent with Sobaih et al., ^24^ who find that transformational leadership significantly enhances organizational performance across various sectors, and emphasize the role of employee empowerment and innovative work climates in translating leadership into performance outcomes.

From a DCT perspective, the importance of transformational leadership lies in its capacity to mobilize change and renew organizational processes in response to evolving demands. ^12^ In regional-owned enterprises, leaders who encourage learning, initiative, and continuous improvement help the organization remain responsive and better positioned to sustain performance. This indicates that transformational leadership contributes not only as an internal capability under RBV, but also as a catalyst for organizational renewal under DCT.

In this study, the strong role of individualized consideration helps explain why dimensions such as company reputation and sales growth are closely associated with better leadership. Employees who feel valued and supported are more likely to deliver higher-quality services, generate positive customer experiences, and sustain productive relationships with stakeholders. These outcomes directly feed into improved reputation and enhanced market performance.

For regional-owned enterprises, where organizations must navigate diverse business lines and complex stakeholder environments, transformational leadership becomes a critical driver of integration and alignment. Leaders who combine inspirational motivation with individualized consideration can mobilize staff to embrace change, adopt new processes, and pursue continuous improvement, which in turn enhances operational excellence and financial results.

### 5.6 The mediating role of business strategy in the relationship between corporate governance and business performance

The indirect effect results show that business strategy significantly mediates the relationship between corporate governance and business performance, with a path coefficient of 0.113 and a p-value of 0.006. This finding indicates that corporate governance does not improve business performance only through a direct pathway, but also through its ability to shape and strengthen business strategy. In other words, stronger governance practices contribute to better performance partly because they provide the structural and procedural foundation for more effective strategic formulation and implementation.

From the perspective of RBV, corporate governance can be understood as an internal organizational capability that helps firms organize, protect, and deploy their resources in a more effective manner. ^11, 63^ Governance mechanisms such as transparency, accountability, fairness, responsibility, and board independence create the internal conditions necessary for disciplined decision-making and strategic alignment. These governance attributes enhance the organization's ability to formulate strategies that are coherent, value-creating, and difficult to imitate, which in turn improves business performance. In this sense, business strategy functions as the mechanism through which governance-based resources are transformed into tangible organizational outcomes.

This mediating relationship is also consistent with DCT, which emphasizes that organizations must not only possess resources, but also continuously configure and reconfigure them in response to environmental change. ^12^ Corporate governance, by itself, may provide oversight and control, but its performance effect becomes stronger when it is translated into strategic actions such as innovation, organizational flexibility, and effective resource allocation. Thus, business strategy serves as the operational channel through which governance supports adaptation and strategic renewal. For regional-owned enterprises operating in complex and regulated environments, this is particularly important because governance quality must be converted into adaptive strategic choices if it is to produce higher levels of financial, operational, and reputational performance.

Overall, the findings demonstrate that business strategy plays an important intervening role in converting corporate governance into improved business performance. The result strengthens the argument that governance should not be viewed merely as a compliance instrument, but as a strategic organizational capability whose value is realized more fully when it informs innovation, flexibility, and resource management. For regional-owned enterprises, this implies that governance reforms will be more effective when they are accompanied by deliberate strategic orientation, so that internal control and accountability mechanisms can be translated into sustainable performance improvements.

### 5.7 The mediating role of business strategy in the relationship between transformational leadership and business performance

The indirect effect results show that business strategy significantly mediates the relationship between transformational leadership and business performance, with a path coefficient of 0.063 and a p-value of 0.035. This finding suggests that transformational leadership contributes to improved business performance not only through its direct influence, but also through its capacity to shape more effective business strategies. In other words, transformational leaders enhance performance partly by fostering strategic orientations that are more innovative, adaptive, and responsive to organizational needs and environmental demands.

From the RBV perspective, transformational leadership can be understood as a valuable intangible organizational resource that enhances the effective use of human and organizational capabilities. ^11, 63^ Through idealized influence, inspirational motivation, intellectual stimulation, and individualized consideration, transformational leaders create the internal conditions needed for strategic coherence and commitment. These leadership qualities help mobilize employees, encourage creative thinking, and align organizational efforts with long-term goals. In this sense, business strategy serves as the mechanism through which leadership-based resources are translated into improved organizational performance.

This mediating relationship is also in line with DCT, which emphasizes the importance of continuously adapting and reconfiguring organizational resources in response to changing conditions. ^12^ Transformational leadership by itself may inspire and motivate organizational members, but its contribution to performance becomes stronger when it is reflected in strategic actions such as product and service innovation, organizational flexibility, and effective resource allocation. Business strategy therefore functions as the operational channel through which transformational leadership is converted into adaptive and performance-enhancing organizational behaviour. For regional-owned enterprises, where leaders must balance commercial goals with public responsibilities, this strategic translation is especially important because leadership effectiveness depends not only on inspiration but also on the ability to direct change in a structured and purposeful way.

Overall, the findings confirm that business strategy plays an important mediating role in translating transformational leadership into better business performance. The result reinforces the view that leadership should not be understood only as a direct driver of performance, but also as a strategic capability whose value is realized through the formulation and implementation of adaptive business strategies. For BUMD *Aneka Usaha*, this implies that transformational leaders will be more effective when their leadership behaviours are accompanied by strategic actions that promote innovation, flexibility, and disciplined resource management.

### 5.8 The mediating role of business strategy in the relationship between transformational leadership and business performance

The results show that government policy significantly moderates the relationship between corporate governance and business performance, with a path coefficient of 0.161 and a p-value of 0.039. H8 is therefore supported. This indicates that supportive government policy amplify the positive effect of corporate governance on business performance.

Contingency Theory provides a useful lens for interpreting this finding. ^35^ The theory posits that no single organizational practice or structure is universally effective; instead, effectiveness depends on its fit with situational factors. In this study, corporate governance represents an internal mechanism, whereas government policy functions as an external contingency factor. The beneficial impact of governance on performance is thus contingent upon the policy environment in which regional-owned enterprises operate. From an RBV perspective, corporate governance can also be viewed as an internal organizational capability whose value is more fully realized when supported by favourable external conditions. ^63^ In addition, from a DCT perspective, a supportive policy environment helps organizations translate governance quality into more adaptive and responsive organizational actions. ^12^

In a conducive policy environment, characterized by consistent regulations, clear oversight mechanisms, and incentives for good governance, internal governance practices are more easily translated into performance improvements. For example, policies that promote electronic procurement, transparency, and anti-corruption allow enterprises with strong accountability and transparency to leverage these systems to enhance efficiency, reduce fraud risks, and improve profitability and reputation. Likewise, policies that encourage investment in strategic sectors enable independent boards to make objective, long-term decisions that optimize financial returns and align with regional development goals.

In this context, government policy acts as a catalyst rather than a substitute. It does not replace the need for robust internal governance but strengthens its impact by creating an environment in which good governance practices can generate maximum value.

### 5.9 The moderating role of government policy in the relationship between transformational leadership and business performance

Government policy also moderates the relationship between transformational leadership and business performance, with a path coefficient of 0.115 and a p-value of 0.025, thereby supporting H7. This indicates that a supportive policy environment enhances the positive impact of transformational leadership on organizational performance.

Transformational leadership creates an internal climate conducive to change, innovation, and high performance. However, the extent to which this leadership can be translated into concrete performance outcomes depends on the external constraints and opportunities defined by government policy. Drawing again on Contingency Theory, transformational leadership is not universally effective in all environments. ^35^ Its success is contingent upon situational conditions, such as regulatory flexibility, autonomy in decision-making, and the availability of incentives for innovation. In a rigid, highly bureaucratic, or unstable policy environment, even highly inspiring leaders may find it difficult to implement strategic initiatives. Conversely, when policies provide room for experimentation, decentralization, and performance-based incentives, transformational leaders can fully leverage their influence to drive organizational change. ^71^

From an RBV perspective, this finding also suggests that the value of transformational leadership as a strategic organizational asset becomes stronger when external conditions are supportive. ^63^ Likewise, from a DCT perspective, supportive policies create room for leadership-driven renewal and performance improvement. ^12^

For regional-owned enterprises, this interplay is particularly visible. When local governments introduce policies promoting digitalization, service innovation, or performance-based budgeting, transformational leaders can use these frameworks as platforms to articulate a compelling vision, mobilize employees, and implement change. This, in turn, improves key dimensions of business performance such as operational excellence and customer satisfaction.

In practical terms, government policy acts as an enabler that allows transformational leadership to be expressed in concrete organizational practices. By aligning regulatory frameworks with the need for innovation, flexibility, and accountability, policymakers create conditions where transformational leaders can translate their vision and relational capital into measurable performance gains.

### 5.10 The moderating role of government policy in the relationship between business strategy and business performance

Finally, the results show that government policy significantly moderates the relationship between business strategy and business performance, with a path coefficient of 0.168 and a p-value of 0.040. H10 is therefore supported. This suggests that the effectiveness of business strategy in enhancing performance is strengthened when it is implemented within a supportive policy environment.

In line with Contingency Theory, strategic success is not determined solely by internal choices but also by the alignment between those choices and external conditions. ^35^ For regional-owned enterprises, strategies focused on innovation, market expansion, or diversification must be consistent with regional development plans, sectoral regulations, and fiscal policies. From an RBV perspective, this finding indicates that the performance value of business strategy depends on how effectively internal capabilities can be deployed under prevailing external conditions. ^63^ From a DCT perspective, supportive policies help strategies remain adaptive and actionable as organizations respond to shifting demands. ^12^

Supportive government policy, such as incentives for innovation, simplified licensing processes, or programs to promote local products, can magnify the positive impact of strategy on performance. Conversely, misaligned or restrictive policies can dampen the effectiveness of even well-designed strategies.

Overall, the findings highlight that business strategy and government policy must be viewed as interdependent rather than separate domains. Strategic planning in regional-owned enterprises should be closely attuned to the evolving policy environment to maximize performance outcomes. A supportive policy framework acts as a reinforcing mechanism that allows strategic initiatives to generate stronger and more sustainable improvements in business performance. ^49^

## 6. Conclusion

This study examined how corporate governance and transformational leadership shape business strategy and business performance in regional-owned enterprises in Indonesia, while also considering the moderating role of government policy. The empirical results show that corporate governance and transformational leadership have positive and significant effects on business strategy and business performance. Business strategy also has a positive and significant effect on business performance and serves as an important mediating mechanism through which corporate governance and transformational leadership are translated into superior organizational outcomes. In addition, government policy significantly strengthens the effects of corporate governance, transformational leadership, and business strategy on business performance. Taken together, these findings show that business performance in BUMD is shaped not only by direct internal capabilities, but also by the organization’s ability to channel those capabilities into adaptive and well-aligned strategic action within a supportive policy environment.

Theoretically, this study contributes by integrating RBV, DCT, and Contingency Theory in explaining business performance in regional-owned enterprises. From the RBV perspective, corporate governance and transformational leadership represent valuable internal capabilities that support organizational effectiveness. From the DCT perspective, these internal capabilities do not automatically produce superior performance unless they are translated into strategic responses that enable adaptation, innovation, and effective resource reconfiguration. In this study, business strategy functions as that strategic mechanism linking internal organizational strengths to performance outcomes. At the same time, Contingency Theory helps explain that the effectiveness of these internal capabilities and strategic actions is conditioned by the external policy environment. This integrated perspective extends the understanding of how governance, leadership, strategy, and policy interact in shaping performance in public enterprises operating in complex institutional contexts.

Practically, the findings imply that BUMD management should not rely solely on formal governance structures or leadership qualities in isolation. Instead, governance and transformational leadership need to be accompanied by business strategies that are flexible, innovation-oriented, and supported by effective resource allocation. Strengthening transparency, accountability, board independence, and leadership behaviours that encourage vision, motivation, intellectual stimulation, and individual development can help BUMD formulate and implement better strategies that improve financial, operational, market, and reputational performance. For policymakers, the results underline the importance of creating coherent and supportive regulatory frameworks that reinforce, rather than constrain, strategic adaptation and organizational renewal in regional-owned enterprises.

This study has several limitations that should be acknowledged. First, the use of a cross-sectional survey design limits the ability to draw strong causal conclusions and does not fully capture how governance, leadership, strategy, and performance may evolve over time. Second, the reliance on self-reported responses from directors may introduce perceptual bias, despite the respondents’ strategic roles in the organizations. Third, the analysis is restricted to BUMD Aneka Usaha in Indonesia, which means that the findings should be generalised to other types of regional-owned enterprises, sectors, or national contexts with caution. Fourth, although the model captures key internal and external factors, it does not include other potentially relevant variables such as digital innovation, organizational culture, stakeholder engagement, or broader institutional capacity. In addition, government policy is measured perceptually rather than through objective regulatory or policy indicators. Future studies could therefore adopt longitudinal or panel designs, incorporate mixed-method approaches that combine quantitative modelling with qualitative case studies, and extend the model by adding mediating and moderating variables such as digital transformation, innovation capability, or public accountability mechanisms. Comparative research across regions, sectors, or countries would further enrich understanding of how different institutional and policy configurations shape the interplay between governance, leadership, strategy, and performance in public enterprises.

## Author contributions

Muhammad Willy: Conceptualization; Methodology; Data Curation; Formal Analysis; Investigation; Writing – Original Draft; Project Administration.

Mochammad Al Musadieq: Conceptualization; Methodology; Validation; Supervision, Writing – Review & Editing.

Yusri Abdillah: Conceptualization; Methodology; Validation; Supervision, Writing – Review & Editing.

Nila Firdausi Nuzula: Conceptualization; Methodology; Validation; Supervision, Writing – Review & Editing.

## Ethics statement and informed consent

### Ethics statement

This study involved human participants and was conducted in accordance with the ethical principles of the Declaration of Helsinki. Prior to participation, all respondents were provided with a standardized verbal script explaining the study’s purpose and procedures, confidentiality and data protection, voluntary participation, and the right to refuse or withdraw at any time without consequences. Ethical approval was granted by the Ethics Committee of Universitas Brawijaya (Approval No. 06218/UN10.F0301/B/PP/2025).

## Informed consent

Verbal informed consent was obtained before data collection and was formally witnessed by a member of the research team. Verbal consent was chosen because the study posed minimal risk, data collection was conducted in a manner that did not require physical documentation, and obtaining written consent would have required collecting names/signatures that could introduce direct identifiers and reduce anonymity. No names or direct personal identifiers were collected.

## Data Availability

Repository name: Business Strategy & Performance: Questionnaire and Data. https://doi.org/10.6084/m9.figshare.30815783 ^72^ This project contains the following underlying data:
- Questionnaire (full survey instrument with item codes) - Data Data are available under the terms of the Creative Commons Attribution 4.0 International license (CC-BY 4.0).
