# Supplementary material for: The Effect of Corporate Governance and Transformational Leadership on Business Strategy and Business Performance Moderated by Government Policy
Source: F1000Res. 2026 Apr 20;15:56. Originally published 2026 Jan 14. [Version 2] doi: 10.12688/f1000research.175209.2 (PMC13146469; doi:10.12688/f1000research.175209.2)
Supplement: Peer review history

# Reviewer report: Reviewer response for version 2

**DOI:** 10.5256/f1000research.198522.r477474

**Published:** 2026-05-07

**Authors:** Muhammed Zakir Hossain

**Affiliations:** 1 Department of Business Studies, State University of Bangladesh, Dhaka, Bangladesh; 2 State University of Bangladesh, dhaka, Bangladesh

While the article is scientifically sound overall, here are a few specific suggestions to improve clarity, transparency, and replicability:

1. Include the full survey questionnaire used in the study as an appendix to ensure that future researchers can replicate the data collection method precisely.
2. Provide the SmartPLS model diagram and the model file to allow researchers to reproduce the path analysis step exactly.
3. Share data processing scripts if any data transformations were done prior to analysis (e.g., handling missing data or data normalization), so others can follow the exact steps taken.
4. Clarify the assumptions underlying the statistical methods used (e.g., normality of data, homoscedasticity) to improve transparency in the analysis.

Is the work clearly and accurately presented and does it cite the current literature?

Yes

If applicable, is the statistical analysis and its interpretation appropriate?

Yes

Are all the source data underlying the results available to ensure full reproducibility?

Partly

Is the study design appropriate and is the work technically sound?

Yes

Are the conclusions drawn adequately supported by the results?

Yes

Are sufficient details of methods and analysis provided to allow replication by others?

Partly

Reviewer Expertise:

Corporate Governance, Artificial Intelligence, Accounting, Auditing, Corporate Social Responsibility

I confirm that I have read this submission and believe that I have an appropriate level of expertise to confirm that it is of an acceptable scientific standard, however I have significant reservations, as outlined above.

# Reviewer report: Reviewer response for version 2

**DOI:** 10.5256/f1000research.198522.r476761

**Published:** 2026-05-05

**Authors:** David Minja

**Affiliations:** 1 Department of Public Policy and Administration, Kenyatta University, Nairobi, Nairobi County, Kenya

Approved for indexing.

Is the work clearly and accurately presented and does it cite the current literature?

Yes

If applicable, is the statistical analysis and its interpretation appropriate?

Yes

Are all the source data underlying the results available to ensure full reproducibility?

Yes

Is the study design appropriate and is the work technically sound?

Partly

Are the conclusions drawn adequately supported by the results?

Partly

Are sufficient details of methods and analysis provided to allow replication by others?

Yes

Reviewer Expertise:

NA

I confirm that I have read this submission and believe that I have an appropriate level of expertise to confirm that it is of an acceptable scientific standard.

# Reviewer report: Reviewer response for version 2

**DOI:** 10.5256/f1000research.198522.r476762

**Published:** 2026-04-21

**Authors:** Oscar Radyan Danar

**Affiliations:** 1 Brawijaya University, Malang, Indonesia

Thank you for the revisions made to the manuscript and for the authors’ careful responses to the comments raised in the previous review. The revised version has addressed the issues identified earlier. I only have minor remaining comment concerning the consistency of terminology used throughout the manuscript, which should be checked carefully to ensure uniformity across sections.

 Nevertheless, this is a minor issue and does not affect the overall quality of the article. In my assessment, the article is suitable to proceed for indexing in Scopus.

Is the work clearly and accurately presented and does it cite the current literature?

Yes

If applicable, is the statistical analysis and its interpretation appropriate?

Yes

Are all the source data underlying the results available to ensure full reproducibility?

Yes

Is the study design appropriate and is the work technically sound?

Yes

Are the conclusions drawn adequately supported by the results?

Yes

Are sufficient details of methods and analysis provided to allow replication by others?

Yes

Reviewer Expertise:

Public Governance

I confirm that I have read this submission and believe that I have an appropriate level of expertise to confirm that it is of an acceptable scientific standard.

# Reviewer report: Reviewer response for version 1

**DOI:** 10.5256/f1000research.193172.r457717

**Published:** 2026-02-28

**Authors:** David Minja

**Affiliations:** 1 Department of Public Policy and Administration, Kenyatta University, Nairobi, Nairobi County, Kenya

In the abstract, the findings imply that business strategy was treated as an independent variable. The same variable is treated as a mediator. This is inconsistent with the study topic because business strategy is treated as a dependent variable while corporate governance and transformational leadership are treated as independent variables in the topic. These inconsistencies should be addressed.

  In section 3.0 on methods, the research design used is missing. This should be included.

 Hypotheses testing

 H3- Business strategy has an effect on business performance. From the topic, both are dependent variables. Conceptually, a      DV cannot have an effect on another DV since it has not been manipulated. This hypothesis should therefore be dropped.

 In the discussions, the study should have shown how Dynamic Capabilities theory mentioned in the background informed this study( it’s relevance). The authors only discussed RBV.

 The study demonstrates how it has contributed to the body of knowledge in the application of RBV  and Contingency Theory. However, how the study contributed to Dynamic Capabilities Theory is not clear.

 In the conclusion section, limitations should be adequately discussed

Is the work clearly and accurately presented and does it cite the current literature?

Yes

If applicable, is the statistical analysis and its interpretation appropriate?

Yes

Are all the source data underlying the results available to ensure full reproducibility?

Yes

Is the study design appropriate and is the work technically sound?

Partly

Are the conclusions drawn adequately supported by the results?

Partly

Are sufficient details of methods and analysis provided to allow replication by others?

Yes

Reviewer Expertise:

NA

I confirm that I have read this submission and believe that I have an appropriate level of expertise to confirm that it is of an acceptable scientific standard, however I have significant reservations, as outlined above.

# Response

**Published:** 2026-04-06

**Authors:** Muhammad Willy

**Affiliations:** Faculty of Administrative Scie, Brawijaya University, Malang, East Java, Indonesia

Thank you very much for your constructive comments and thoughtful feedback on our manuscript. The following are the details of the revisions made in response to your comments.

 In the abstract, the findings imply that business strategy was treated as an independent variable. The same variable is treated as a mediator. This is inconsistent with the study topic because business strategy is treated as a dependent variable while corporate governance and transformational leadership are treated as independent variables in the topic. These inconsistencies should be addressed.

**Author response:** Thank you for this valuable comment. We agree that the previous wording in the abstract could create ambiguity regarding the role of business strategy. We have revised the abstract to clarify that business strategy is positioned as an endogenous variable influenced by corporate governance and transformational leadership, and as a mediating variable linking these variables to business performance. The revised wording now presents the variable roles consistently with the study model and title.

 In section 3.0 on methods, the research design used is missing. This should be included.

**Author response:** Thank you for this comment. We have revised Section 3 to explicitly state the research design. The manuscript now clarifies that the study used a quantitative, explanatory design with a cross-sectional survey approach.

 Hypotheses testing

 H3- Business strategy has an effect on business performance. From the topic, both are dependent variables. Conceptually, a DV cannot have an effect on another DV since it has not been manipulated. This hypothesis should therefore be dropped.

**Author response:** Thank you for this important comment. We respectfully clarify that business strategy was not conceptualized as a final dependent variable in the same sense as business performance. In our model, business strategy is specified as an endogenous mediating construct that is influenced by corporate governance and transformational leadership and, in turn, affects business performance. Thus, H3 is retained because it represents the mediating path required in the structural model. To avoid ambiguity, we have revised the manuscript to clarify the role of business strategy as mediating mechanism rather than as a standalone final dependent variable.

 In the discussions, the study should have shown how Dynamic Capabilities theory mentioned in the background informed this study( it’s relevance). The authors only discussed RBV.

 The study demonstrates how it has contributed to the body of knowledge in the application of RBV  and Contingency Theory. However, how the study contributed to Dynamic Capabilities Theory is not clear.

**Author response:** Thank you for this comment. We have revised the Discussion and Conclusion to clarify the relevance and contribution of Dynamic Capabilities Theory in the study.

 In the conclusion section, limitations should be adequately discussed.

**Author response:** Thank you for this comment. We agree that the limitations section needed to be discussed more explicitly. We have therefore revised the conclusion by strengthening the discussion of the study’s limitations, including the cross-sectional design, reliance on self-reported data, the contextual focus on BUMD Aneka Usaha in Indonesia, and the exclusion of other potentially relevant variables. We also clarified how these limitations inform directions for future research.

# Reviewer report: Reviewer response for version 1

**DOI:** 10.5256/f1000research.193172.r457713

**Published:** 2026-02-16

**Authors:** Oscar Radyan Danar

**Affiliations:** 1 Brawijaya University, Malang, Indonesia

I reviewed the manuscript and overall the research method is appropriate, the analysis is presented clearly, and the findings are consistent with the study’s objectives and proposed framework. The topic is relevant for both policy and managerial discussion, and the paper provides a useful empirical contribution.

**Minor comments (non-essential)**

 1. Strengthen the contribution statement in the Introduction:

 At the end of the Introduction, please add 2–3 short sentences that clearly summarize the research gap, the study aim, and the main contributions in one concise paragraph.

 2. Clarify the random sampling procedure:

 Please briefly state the sampling procedure. This will improve transparency and make replication easier.

 3. Improve terminology consistency:

 Please use consistent key terms throughout the manuscript (choose either “government policy” or “government policies” and use it consistently, and keep the organizational context naming consistent).

Is the work clearly and accurately presented and does it cite the current literature?

Yes

If applicable, is the statistical analysis and its interpretation appropriate?

Yes

Are all the source data underlying the results available to ensure full reproducibility?

Yes

Is the study design appropriate and is the work technically sound?

Yes

Are the conclusions drawn adequately supported by the results?

Yes

Are sufficient details of methods and analysis provided to allow replication by others?

Yes

Reviewer Expertise:

Public Governance

I confirm that I have read this submission and believe that I have an appropriate level of expertise to confirm that it is of an acceptable scientific standard.

# Response

**Published:** 2026-04-06

**Authors:** Muhammad Willy

**Affiliations:** Faculty of Administrative Scie, Brawijaya University, Malang, East Java, Indonesia

I reviewed the manuscript and overall the research method is appropriate, the analysis is presented clearly, and the findings are consistent with the study’s objectives and proposed framework. The topic is relevant for both policy and managerial discussion, and the paper provides a useful empirical contribution.

**Author response: **Thank you for your positive and encouraging comments. We appreciate your recognition that the research method is appropriate, the analysis is clearly presented, and the findings are aligned with the study’s objectives and framework. We are also grateful that you consider the topic relevant for both policy and managerial discussion and that the paper offers a useful empirical contribution.

 Minor comments (non-essential)

 1. Strengthen the contribution statement in the Introduction:

 At the end of the Introduction, please add 2–3 short sentences that clearly summarize the research gap, the study aim, and the main contributions in one concise paragraph.

**Author response: **We have strengthened the contribution statement at the end of the Introduction by adding a concise paragraph that explicitly summarizes the research gap, the study aim, and the main theoretical and practical contributions of the study.

 2. Clarify the random sampling procedure:

 Please briefly state the sampling procedure. This will improve transparency and make replication easier.

**Author response:** We have revised the Methods section to clarify the random sampling procedure.

 3. Improve terminology consistency:

 Please use consistent key terms throughout the manuscript (choose either “government policy” or “government policies” and use it consistently, and keep the organizational context naming consistent).

**Author response: **We have revised the manuscript to improve terminology consistency throughout the text.
